# The Role of Integrin Receptor’s α and β Subunits of Mouse Mesenchymal Stem Cells on the Interaction of Marine-Derived Blacktip Reef Shark (*Carcharhinus melanopterus*) Skin Collagen

**DOI:** 10.3390/ijms24119110

**Published:** 2023-05-23

**Authors:** Baolin Ge, Mingjun Wei, Bin Bao, Zhilin Pan, Jeevithan Elango, Wenhui Wu

**Affiliations:** 1 Department of Marine Pharmacology, College of Food Science and Technology, Shanghai Ocean University, Shanghai 201306, China; 13561936983@163.com (B.G.); weimj98@163.com (M.W.);; 2 Department of Biomaterials Engineering, Faculty of Health Sciences, UCAM-Universidad Católica San Antonio de Murcia, Guadalupe, 30107 Murcia, Spain

**Keywords:** blacktip shark collagen, mesenchymal stem cells, integrin, marine biomaterials, extracellular matrix

## Abstract

Marine collagen (MC) has recently attracted more attention in tissue engineering as a biomaterial substitute due to its significant role in cellular signaling mechanisms, especially in mesenchymal stem cells (MSCs). However, the actual signaling mechanism of MC in MSC growth, which is highly influenced by their molecular pattern, is poorly understood. Hence, we investigated the integrin receptors (α_1_β_1_, α_2_β_1_, α_10_β_1_, and α_11_β_1_) binding mechanism and proliferation of MCs (blacktip reef shark collagen (BSC) and blue shark collagen (SC)) compared to bovine collagen (BC) on MSCs behavior through functionalized collagen molecule probing for the first time. The results showed that BSC and SC had higher proliferation rates and accelerated scratch wound healing by increasing migratory rates of MSCs. Cell adhesion and spreading results demonstrated that MC had a better capacity to anchor MSCs and maintain cell morphology than controls. Living cell observations showed that BSC was gradually assembled by cells into the ECM network within 24 h. Interestingly, qRT-PCR and ELISA revealed that the proliferative effect of MC was triggered by interacting with specific integrin receptors such as α_2_β_1_, α_10_β_1_, and α_11_β_1_ of MSCs. Accordingly, BSC accelerated MSCs’ growth, adhesion, shape, and spreading by interacting with specific integrin subunits (α_2_ and β_1_) and thereby triggering further signaling cascade mechanisms.

## 1. Introduction

Collagen is the principal structural protein in the extracellular matrix (ECM) and accounts for more than one-third by weight of the human body’s total protein [1]. It is widely found in the connective tissues, such as skin, bones, tendons, ligaments, cartilage, and cornea of animals [2]. The collagens represent a superfamily of proteins that includes type I, II, III, V, and XI [3] and contain a triple helical domain that can activate and maintain the interaction between cells and the ECM [4]. Previous research demonstrated that collagen is a useful biomaterial in various fields due to its excellent biocompatibility, biodegradability, accessibility, and flexibility [5,6]. Nevertheless, because of the existence of problems, diseases, and religious beliefs, for instance, the application of collagen and collagen-derived products in terrestrial animals is limited [7]. At present, collagen has been isolated from various marine organisms [8,9]. Marine-derived collagen has high biocompatibility, low immunogenicity, easy absorption, and no religious restrictions [10]. Therefore, the biomaterials from marine collagen are a more suitable source than mammal counterparts in medical tissue engineering applications [11].

Efficacious stem cell-based therapies for tissue engineering and regenerative medicine require a biomaterial to improve stem cell preservation in injury and orchestrate tissue repair [12,13,14]. Nevertheless, an adequate number of multipotent osteoprogenitors, such as mesenchymal stem cells (MSCs), is requisite for efficient bone tissue repair [15,16]. Particularly, mesenchymal stem cells interact with their microenvironment that regulates diverse behaviors such as proliferation, migration, and adhesion [17]. Thus, MSCs are frequently seeded on biomaterials with collagen [18], and it is imperative that the research on how potentially feasible collagen biomaterials affect MSCs develop optimal strategies for MSC expansion and bone tissue regeneration. Over the past years, marine collagen-based biomaterials based on tissue engineering strategies have been proposed and developed [19,20].

More recently, our studies demonstrated that the marine-derived blacktip reef shark skin collagen (BSC) has superior physicochemical, structural, and functional properties and could be a viable material for biomaterial fabrication [21]. Unfortunately, it is still unclear whether the BSC influences the cell behaviors of MSCs. Therefore, the future applications of this collagen biomaterial must elucidate the interaction between BSC and MSCs. Despite the effect of BSC on the osteogenic differentiation of MSCs being significant, in this study, we were more interested in some short-term cellular interactions prior to MSC differentiation. We focus on how the BSC affects stem cell proliferation, migration, adhesion, spreading, and ECM remodeling. Moreover, we also investigated the effect of BSC on integrin expression on the surface of MSCs in order to try to reveal the possible profound modulation mechanism in stem cell behavior.

## 2. Results

### 2.1. Cell Proliferation and Viability

**Figure 1:**
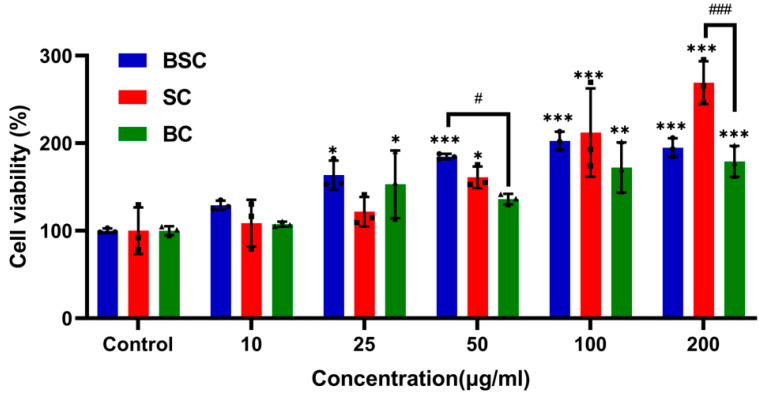
Cell proliferation of MSCs cultured with different concentrations of collagens from blacktip reef shark skin (BSC), blue shark skin (SC), and bovine (BC) for 24 h. The data represent mean ± SD, *n* = 3. *, ** and *** indicate *p* < 0.05, *p* < 0.01, and *p* < 0.001 compared with control, respectively. #, ### indicate *p* < 0.05, *p* < 0.001 are significantly different between the two groups.

In the beginning, we explored the effect of diverse collagen on MSC proliferation in a metrological gradient by CCK-8. The proliferation and viability of MSCs cultured in collagens are shown in Figure 1. After culturing for 24 h, the pattern of MSC proliferation was increased with the addition of increasing concentrations of collagens, but the marine collagen was higher than bovine collagen. However, from 10 μg/mL to 25 μg/mL, SC showed no statistically significant difference compared with the control, and cell viability was not substantially enhanced among three collagens at a 10 μg/mL concentration.

Surprisingly, cells cultured in the presence of 50 μg/mL BSC and 200 μg/mL SC showed a higher proliferative rate compared to bovine collagen. Meanwhile, from 25 μg/mL to 200 μg/mL, BSC had a higher cell proliferation rate than the control, which indicated that BSC has the potential to promote MSC proliferation.

### 2.2. Cell Scratch Wound Healing

**Figure 2:**
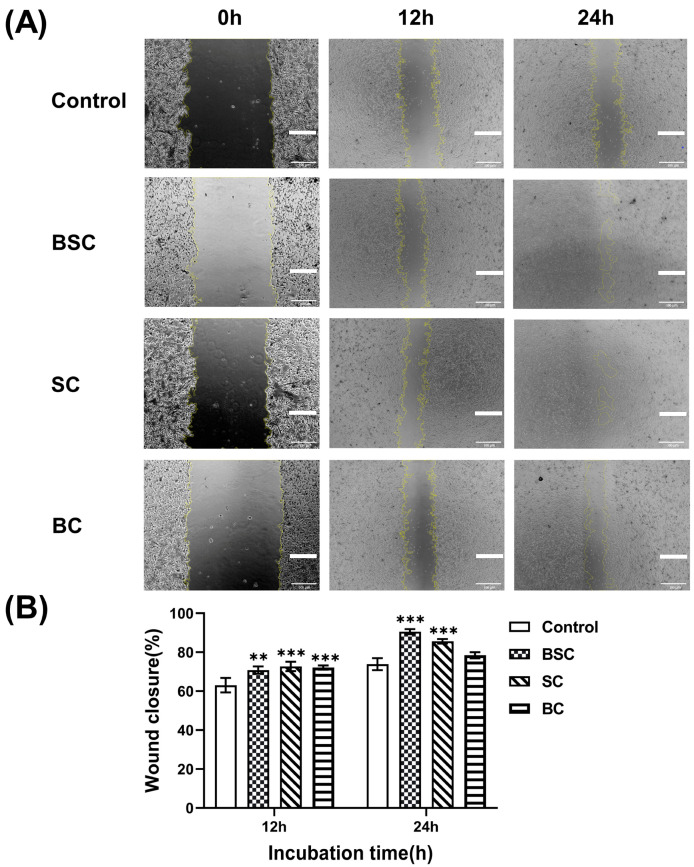
Effect of marine collagen on the scratch wound healing. (**A**) Microscopic images of MSCs treated with different collagens in the scratch assay. The images were captured at 0, 12, and 24 h after incubation. (**B**) Quantitative analysis of the migration area reported as % wound closure. The data represent mean ± SD, *n* = 3. **, and *** indicate *p* < 0.01, and *p* < 0.001 compared with control, respectively. Scale bar: 100 μm.

The scratch assay was performed to observe cell migration, which is an in vitro test for the wound healing process. Cells were cultured in the presence or absence of the different collagens at a concentration of 100 μg/mL and photographed at 0 h, 12 h, and 24 h; the wound closure rate was calculated as described in the Materials and Methods, and the results are shown in Figure 2. The behavior of MSCs indicated that collagen potently induced cell migration by the sides of the wound, gradually filling the gap within 12 h (Figure 2A). Cell migration across the scratch wound area was significantly enhanced when treated with the marine collagen group (BSC 12 h: 70.82 ± 1.9, BSC 24 h: 90.56 ± 1.3; SC 12 h: 72.72 ± 2.4, SC 24 h: 85.63 ± 1.14) compared to the control group (12 h: 63.05 ± 3.7; 24 h: 73.89 ± 3.1) within 24 h, as quantified in Figure 2B. Conversely, by treatment with bovine collagen (12 h: 72.12 ± 1.09; 24 h: 78.47 ± 1.59), the differences in cell migration rates were not remarkable at 24 h, whereas significant effects on wound closure were observed 12 h after treatment compared with the control group.

### 2.3. Cell Adhesion and Spreading

**Figure 3:**
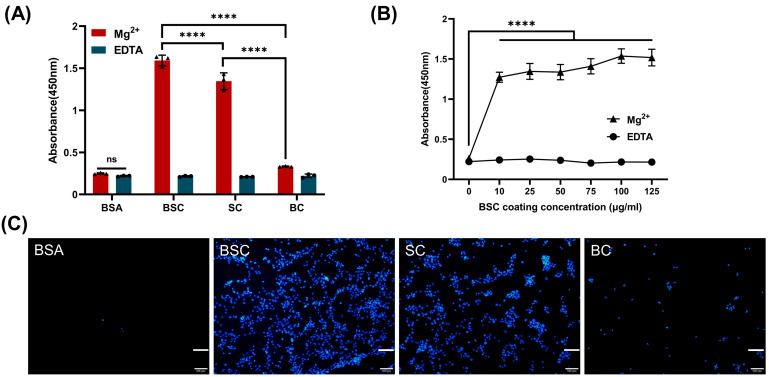
Cell adhesion capacities of marine collagens. (**A**) High-binding polystyrene 96-well plates were coated with BSC, SC, and BC at 1 μg protein per well. MSCs were seeded at a density of 8000 cells per well for 6 h. Cell adhesion assays indicated that BSC and SC displayed superior capacity in anchoring MSCs; ns (not significant) (*p* > 0.05) indicating non-significant differences in the presence of 5 mM Mg^2+^ or 5 mM EDTA in the BSA group. (**B**) MSCs attachment on increasing concentrations of BSC coating 96-well plates. (**C**) The cell nuclei of MSCs were visualized using DAPI in cell adhesion assays (diagram (**A**)). The data represent mean ± SD, *n* = 3. **** indicate *p* < 0.0001 compared with each other in any groups (**A**) or between any BSC coating concentration and BSA (**B**). Scale bar: 100 μm.

The cell adhesion in the presence of Mg^2+^ (integrin-mediated) and EDTA (non-divalent cation specific) was studied with the CCK-8 method. The results indicated that the OD values generated from the CCK-8 treatment in different collagen coating groups with the addition of Mg^2+^ were statistically different. Marine collagen BSC and SC promoted significantly higher cell adhesion than the bovine collagen and BSA control group (Figure 3A). It can be observed that all cell adhesion on marine collagen coating is Mg^2+^-dependent, and the adhesion of MSCs on different materials with added EDTA did not significantly differ from the BSA control group with Mg^2+^. The results indicated that after the removal of Mg^2+^ cations by chelation with EDTA or blocking with BSA, the MSCs could not adhere to the bottom of the plate, and the binding rate of the cells to the substrates was achieved through Mg^2+^. This phenomenon suggests that integrins associated with metal ion-dependent adhesion sites mediate cell adhesion between BSC or SC and MSCs. As anticipated, BSC-coated surfaces displayed significantly higher cell attachment with increased substrate concentration, and the process was affected by the affinity of integrin to collagen (Figure 3B). The promotion of BSC and SC to cell adhesion was also confirmed by the fluorescent cell images (Figure 3C).

**Figure 4:**
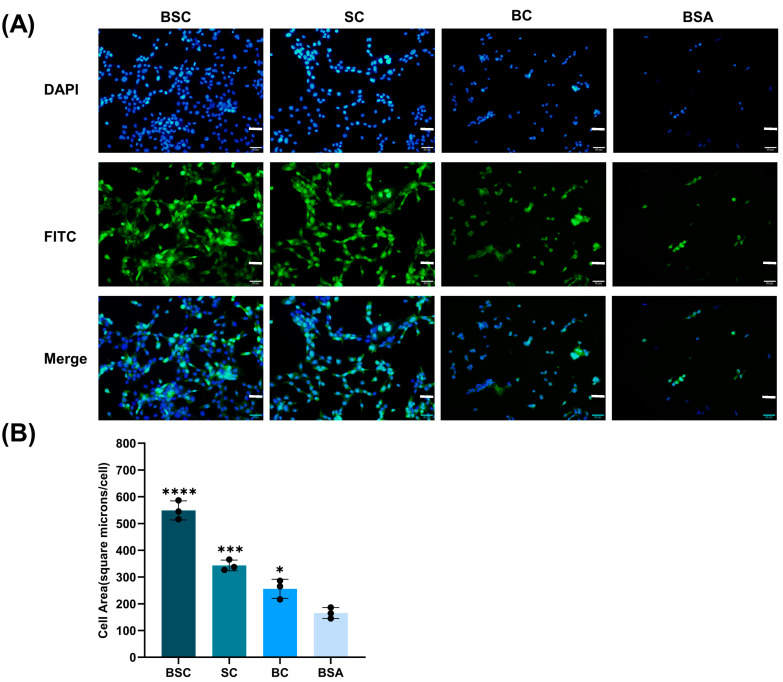
Effect of treating different collagens on MSCs spreading. (**A**) High-binding polystyrene 96-well plates were coated with BSC, SC, and BC at 1 μg protein per well. MSCs were seeded at a density of 25,000 cells per well and allowed to spread for 4 h. The attached cells were fixed with 4% paraformaldehyde, stained with FITC (green) and DAPI (blue), and imaged by fluorescence microscopy. (**B**) Cell area quantification for MSCs on different collagen coating wells. The data represent mean ± SD, *n* = 3. *, *** and **** indicate *p* < 0.01, *p* < 0.001, and *p* < 0.0001 compared with a blank group (BSA), respectively. Scale bar: 50 μm.

Cells were allowed to spread for 4 h, and cell morphological analysis was performed to determine if the BSC could promote cell spreading (Figure 4). This assay was also conducted in the presence of Mg^2+^ as the cells were anchored to the collagen. MSCs adhered to the collagen coatings, and their morphology and number were affected by the presence of different collagen (Figure 4A). MSCs possessed a polygonal morphology on BSC and SC coatings. Cells of rounded and polygonal morphology were observed in the BC coating, and the rounded morphology in BSA control indicated that it did not support cell spreading. In contrast, there were significant differences between BC and the negative BSA control in cell area by quantitative analysis (Figure 4B). The BSC significantly increased the MSCs spreading area to 549.15 ± 35.59 μm^2^ compared to the control (165.24 ± 20.51 μm^2^). Similarly, both the other collagen-coated wells showed cell areas of 343.19 ± 19.95 μm^2^ and 256.07 ± 35.84 μm^2^ for SC and BC, respectively, and the results showed a high degree of cell spreading for all surfaces.

### 2.4. Effect of BSC in the Extracellular Matrix

**Figure 5:**
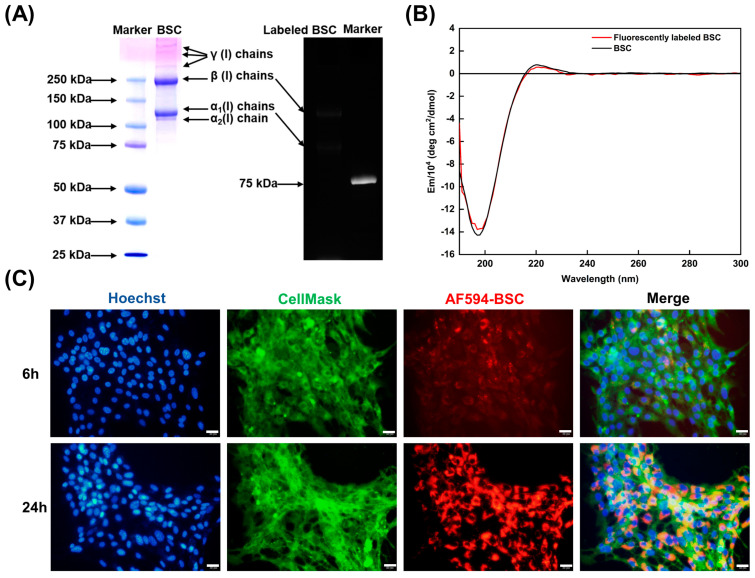
Analysis of a functionalized BSC molecule probe in the extracellular matrix of MSCs. (**A**) SDS–PAGE analysis of AF-594 succinimidyl ester labeled BSC stained with Coomassie brilliant blue R-250 solution and captured with the molecular imager. (**B**) Fluorescently labeled BSC demonstrates a typical maximum absorption band at around 222 nm in the circular dichroism (CD) spectra, indicative of a triple helical structure. (**C**) MSCs incorporate exogenously labeled BSC (red color) into an extracellular collagenous network. Cell membranes (green color) were stained with the CellMask Green plasma membrane stain and nuclei (blue color) with Hoechst 33,342, respectively. Scale bar: 20 μm.

As the molecular structure of collagen is closely related to its biological activity, protein functionalization may disrupt the protein conformation. It was important to confirm whether the functionalized BSC molecules probe (AF594-BSC) retained their secondary structure and triple helical structure. The protein molecular pattern of BSC is similar to our previous study, and the fluorescence image of AF-594 succinimidyl ester labeled BSC showed luminescent α and β chains of type I collagen (Figure 5A). CD spectra of fluorescently labeled BSC were analyzed relative to BSC and exhibited a preeminent positive band at 222 nm and a negative band at 198 nm, which is typical of a collagen triple helix (Figure 5B). The above results showed that the AF594-BSC has the complete collagen triple helix; AF594 did not change the molecular weight and protein secondary structure of BSC, which can be used for molecular tracking in the cell system.

Before the observation, MSC membranes and nuclei were stained green and blue, respectively, allowing for live monitoring of living cells using fluorescent microscopy (Figure 5C). The formation and development of the ECM were observed after the cells were cultured for 6 h and 24 h with the addition of AF594-BSC. Exogenously labeled BSC was only internalized slightly by MSCs and translocated to ECM networks within 6 h. However, after 24 h of cell culture in AF594-BSC, the amount of labeled collagen in the ECM was significantly higher compared to the 6 h incubation. The binding of BSC to the cell membrane is gradually integrated into the newly constructed ECM of MSCs and becomes a component of the extracellular substance.

### 2.5. Effect of Marine Collagen on MSCs Integrin Expression

**Figure 6:**
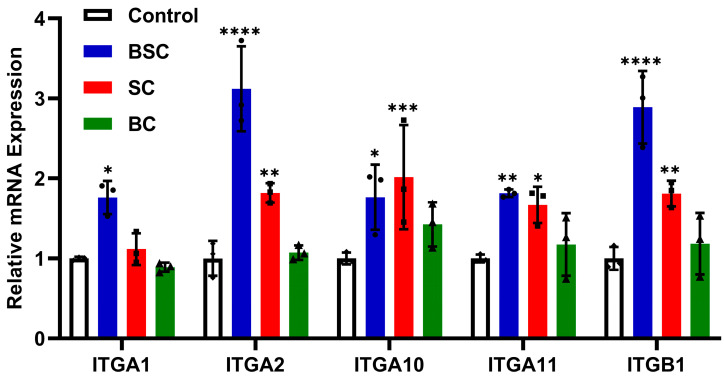
Relative mRNA expression levels of integrin receptor subunits were evaluated by qRT-PCR. ITGA1 (integrin α_1_), ITGA2 (integrin α_2_), ITGA10 (integrin α_10_), ITGA11 (integrin α_11_), and ITGB1 (integrin β_1_) expression in mouse mesenchymal stem cells (MSCs) on different collagens after 24 h of culture. The data represent mean ± SD (*n* = 3) and were analyzed with two-way ANOVA by Tukey’s multiple comparisons tests. *, **, *** and **** indicate *p* < 0.05, *p* < 0.01, *p* < 0.001, and *p* < 0.0001 compared with control, respectively.

**Figure 7:**
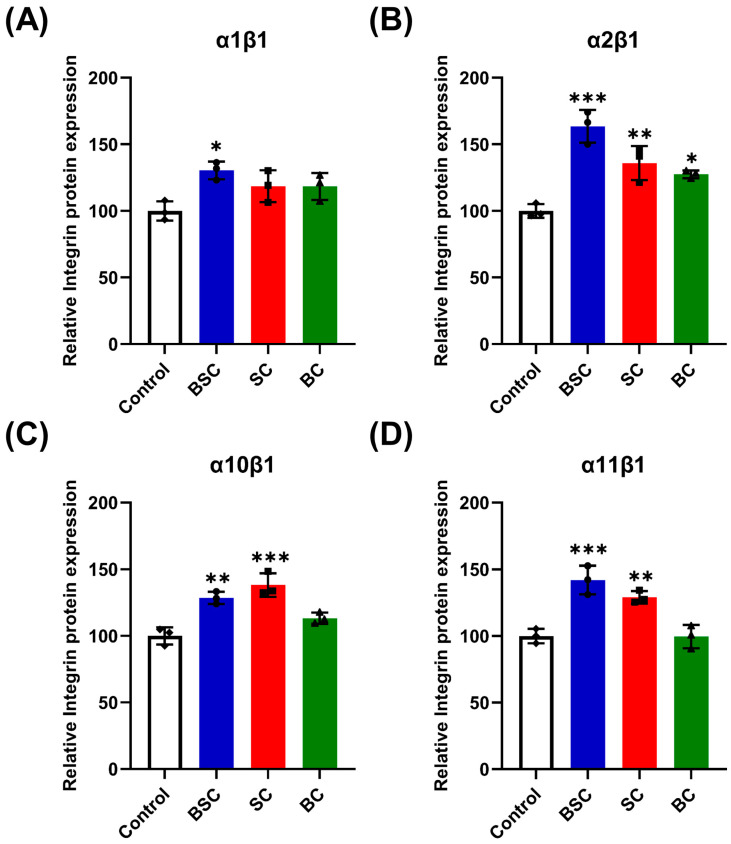
Relative protein expression of four kinds of collagen-binding integrin receptor subunits for MSCs cultured with marine collagens for 24 h. Integrin α_1_β_1_ (**A**), α_2_β_1_ (**B**), α_10_β_1_ (**C**), and α_11_β_1_ (**D**) were quantified via ELISA assay using treated MSCs supernatant. The data represent mean ± SD, *n* = 3. *, **, and *** indicate *p* < 0.05, *p* < 0.01, and *p* < 0.001 compared with control, respectively.

There are many different integrins on the surface of MSCs [22]; integrin receptors that directly bind to collagen, such as α_1_β_1_, α_2_β_1_, α_10_β_1_, and α_11_β_1_, were examined. To determine whether the integrins are involved in cell behavior in MSCs grown on the BSC coating, the mRNA expression of integrins was evaluated by qRT-PCR (ITGA1, ITGA2, ITGA10, ITGA11, and ITGB1) and ELISA after 24 h incubation. Real-time PCR analysis of integrin mRNA showed a relative increase in the number of integrin receptor subunits in MSCs cultured on BSC collagen fibrils (Figure 6), and each group has statistical significance compared to the control. In particular, the mRNA expression of subunits α_2_, α_11_, and β_1_ drastically increased (Figure 6), indicating an elevated assemblage of these α subunits and β1 subunits on the surface of the MSCs. Meanwhile, protein expression of integrin α_2_β_1_ (Figure 7C) and α_11_β_1_ (Figure 7D) showed similar statistical differences. While we observed no difference with the subunit α_1_ expression within 24 h, other subunits were all upregulated on SC substrates (Figure 7). According to our results, subunit α_10_ expression in MSCs cultured on SC collagen fibrils was significantly higher than in cells cultured on BSC fibrils and BC fibrils (Figure 6), and similar differences in integrin receptor expression shown by the ELISA test (Figure 7B).

While there was a relative increase effect in the bovine collagen group, statistical significance was not attained when compared to the control (Figure 6). However, ELISA analyses of α_2_β_1_ receptor in the cell membrane indicated a significant increase after 24 h culture on the BC surface (Figure 7B). In summary, compared with bovine collagen and the control, MSCs cultured on marine collagen showed significantly higher integrin receptors expression for α_2_β_1_, α_10_β_1_, and α_11_β_1_. These data demonstrate that adhering to BSC and SC matrix stimulates integrin expression during the cell behavior regulation of MSCs; bovine collagen does not seem to promote integrin higher expression to affect stem cell behavior.

## 3. Discussion

We explored the marine-derived BSC interactions with mesenchymal stem cells and the influence of cell surface receptors-integrin expression in the present study. The focus of this study is to observe whether collagen interacts with integrin located on the cell surface [23,24,25] and drives specific cellular behaviors, including proliferation, migration, adhesion, and extracellular matrix remodeling [26]. According to our study, it was found that marine collagen BSC and SC can efficiently promote MSC proliferation, while mammal collagen BC also had similar results. Compared with the cell viability of the bovine collagen, the marine collagen was more beneficial for the growth of MSCs, with the highest cell proliferation of more than twice that of the control. Our early study found that blue shark skin collagen effectively increased the proliferation rate of differentiated mouse bone marrow-mesenchymal stem cells [27]. The one reason that BSC may promote MSC proliferation relates to its amino acid composition; it has been reported that collagen-derived dipeptide proline-hydroxyproline (Pro-Hyp) promoted cell proliferation and hyaluronic acid synthesis in human dermal fibroblasts [28]. Proline and hydroxyproline are the main components in BSC [21], and proline is a major amino acid involved in the synthesis of polyamines, which are key regulators of cell proliferation [29]. Another reason may be related to collagen-binding integrin. Marine collagen BSC promotes the expression of integrin on the cell surface by enhancing its affinity with integrin, causing intracellular growth factor signal transmission and accelerating the progression of cells from the G1 phase to the S phase of the cell cycle [30,31], resulting in the proliferation of MSCs.

The migration and proliferation of mesenchymal stem cells play a pivotal role in the different stages of bone healing. MSCs migrate to the defect site and later proliferate and differentiate into osteoblasts and chondrocytes to promote bone formation [32,33]. Cell migration assay results demonstrated that BSC accelerated scratch wound healing by increasing the migratory rates of MSCs. This might be attributed to the abundant amino acid residues in BSC providing a nutritional environment to induce MSC migration [9]. In comparison, both marine collagen and animal collagen had an outstanding capacity to induce MSC migration. The behavior of MSCs in this study indicates cells were first observed migrating to the scratch gap area, followed by enhanced cell proliferation, and the results are similar to those obtained in L929 fibroblasts by earlier research [34,35]. Moreover, the 𝛼_2_𝛽_1_, 𝛼_4_𝛽_1_, 𝛼_5_𝛽_1_, and 𝛼_11_𝛽_1_ integrins play a key role in recruiting these MSCs to the site of injury [36]. Kolambkar et al. demonstrated that 𝛼_2_𝛽_1_ integrin activation by GFOGER-like sequences in collagens, a peptide sequence in the collagen triple helix, increased MSC migration in vitro [37].

Cell adhesion and spreading analysis suggested that marine collagen had a better capacity to adhere and anchor MSCs than bovine collagen and BSA control. Subsequently, in the determinization of the BSC adhesion effect of gradient concentration, BSC wells retained more MSCs compared to BSA blocking wells, further confirming the increased concentration could lead to the superior cell adhesion capacity of the BSC surfaces. Short-term cell morphological observations suggested that the wells coated with marine collagen or bovine collagen all demonstrated their spreading capacity. It is evident from the cell area measurement data that MSCs possessed a large cell spread area on the BSC and SC surfaces. We can determine that MSCs are directly bonded to the BSC surface by integrin receptors and influence cell morphology. Since the tests were carried out in the absence of serum in the cell media, the possibility of bridging between the collagen and the cells by serum proteins such as fibronectin and vitronectin was eliminated [38,39,40]. Indeed, the reasons for the distinct adhesion and spreading capacity of MSCs on the three collagen surfaces might be a consequence of differences in the exposure of cell adhesion sequences in these substrates [41]. Integrins bind to a series of motifs within collagen, which often contain a specific GxOGEx’ similar motif where x is a hydrophobic residue and x’ is usually arginine (R) [42,43]. For instance, the GFOGER motif we mentioned above has been identified as what remains the highest-affinity triple helical ligand for 𝛼2𝛽1 [44]. Furthermore, GLOGER was proved to be a higher affinity selective ligand for 𝛼1𝛽1 [45], and other similar sequences occur in specific loci within the D-periods of collagen type I fibers [43]; they are generally GAOGER or GMOGER, a previously proposed recognition sequence [46,47]. Besides, the BSC and SC retained their native triple helix structures upon the surface of plates resulting in a greater higher affinity towards cell integrin receptors in comparison to the bovine collagen. Therefore, BSC showed greater adhesion activity due to cell adhesion to GxOGEx’ motifs being dependent upon a complete triple-helical conformation [41]. Analyses of MSC morphology showed there might be some cell signaling stimulated by collagen that led to the cell spread area increasing. Hence, integrin-mediated cell engagement with BSC can induce signaling cascades that control cell spreading processes. Overall, these results demonstrate that integrin-collagen interactions can profoundly influence MSCs’ adhesion, shape, and spreading.

We have developed a functionalized BSC molecule probe for visualizing and tracking collagen molecules in live cell culture. Early [48] and recently [49], similar work also provided useful information for our research. There were many finely punctate distributions of AF594-BSC on the cell surface within 6 h and fluorescent collagens were generally not evident in the extracellular matrix at this time. By 24 h, an intricate extracellular matrix network developed at the surface of MSCs under cellular control. The reassembly of BSC in the extracellular matrix was intimately associated with 𝛼2𝛽1 integrin-mediated cell-ECM interactions [50], although fibronectin may also be involved in extracellular matrix remodeling [51]. A previous study indicated that collagen deposition was dependent on fibronectin and enhanced by integrins 𝛼2𝛽1 and 𝛼11𝛽1 [52]. Nevertheless, our current observations here suggest that BSC can be localized in the extracellular matrix within the cellular microenvironment, where collagen-integrin binding is present at those sites with strong fluorescence. Hence, collagen interactions with cell surface integrin receptors control many cell processes, including modulating cell adhesion, cell migration, and ECM assembly or remodeling [53].

Based on these results, we speculate that the specific cell behavior of MSCs may be induced by the collagen-integrin interplay and the intracellular signaling cascades. We report that MSCs expressed high amounts of integrin α_1_β_1_, α_2_β_1_, α_10_β_1_, and α_11_β_1_ observed by ELISA (protein level) and qRT-PCR (mRNA level) in the presence of BSC. Furthermore, we also found that the expression of three integrins significantly increased at the mRNA or protein level in MSCs grown on the BC treatment. The present work seems to indicate that integrin α_2_β_1_ and α_11_β_1_ were the key factors for the modulation of cellular behavior due to the expression in MSCs being statistically significantly higher. The α_2_β_1_ and α_11_β_1_ integrins that bind to collagen play a pivotal role in the survival and proliferation of MSCs [36]. Moreover, integrin α_2_β_1_ has been demonstrated to improve MSC proliferation in vitro [54]. In contrast, integrin α_1_β_1_ has often been reported to play a major role as a modulator of mesenchymal proliferation and differentiation [55]. Integrin α_10_β_1_ shows a distribution that is restricted to mesenchymal stem cells and chondrocytes [56] compared to the α_1_β_1_ and α_2_β_1_ being widely expressed on cells in contact with basement membranes [22,55,57]. The expression of integrin α_10_ can be upregulated by fibroblast growth factor 2 [56,58] to control the migration of MSCs [59]. Whereas Wenke et al. proved the expression of integrin α_10_ plays a role in the migration of malignant melanoma cells [60]. It is interesting to note that integrin α_10_β_1_ was extremely significantly upregulated on SC substrates, which might lead to promoting the migration of MSCs via the binding of integrin and collagen. Similarly, the α_11_β_1_ as a multifunctional integrin was also involved in cell migration [61,62], and the integrin α_11_ I domain recognizes the triple-helical GFOGER sequence present in type I collagen as well as the GLOGER motif [46,63]. In general, integrin α_2_β_1_ and α_11_β_1_ overexpression can promotes the adhesion of type I collagen to cells [42]. However, the function of integrins might be regulated by different factors such as expression level on the cell surface and conformation of heterodimers (expressed in active form or inactive form). At the same time, the activation and interaction of integrins by collagen are highly influenced by the amino acid composition and denaturation temperature of shark collagen and bovine collagen.

**Figure 8:**
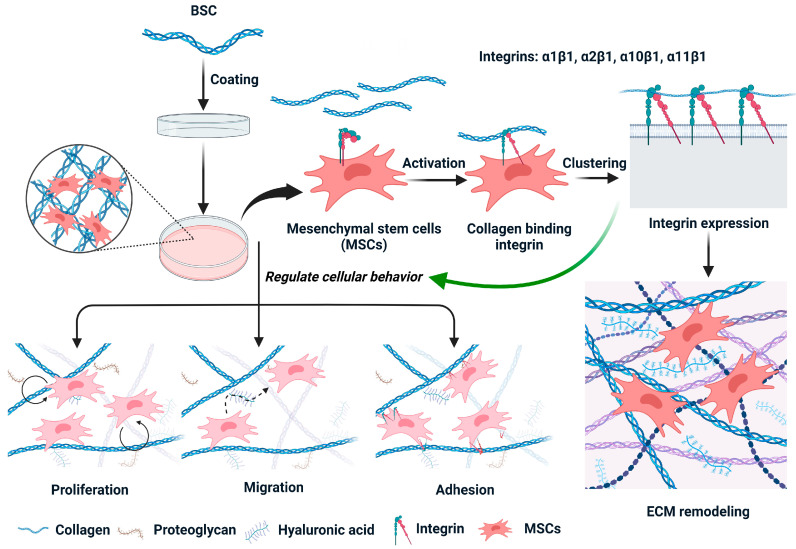
Marine-derived BSC binding integrin can be used to regulate cell behavior for improved tissue regeneration.

Based on previous research and the present results, we conclude that blacktip reef shark skin-derived collagen-integrin interplay might be involved in the regulation of cellular behavior, including migration, proliferation, adhesion, morphogenesis, and ECM remodeling (Figure 8). In the present study, the better activity of marine collagen compared to bovine collagen in MSCs growth, wound closure, and integrin’s interaction could be due to the partial degradation of shark collagen during cell culture at 37 °C since the denaturation temperature of shark collagen, in general, was less than 30 °C [27]. It is well evidenced that the specific amino acid residues such as Gly-Pro-Hyp released from denatured collagen could improve the biological response of collagen. Although our findings cannot fully demonstrate the profound mechanisms that BSC-integrin binding modulates cell behavior in the cellular microenvironment, future studies could further elucidate the in-depth signaling mechanism of BSC, and it would be a valuable biomaterial candidate for regenerative medicine and therapy.

## 4. Materials and Methods

### 4.1. Chemicals and Materials

Blacktip reef shark skin-derived collagen (BSC) and blue shark skin-derived collagen (SC) were extracted and characterized according to our previous reports [21,27]. Bovine collagen (BC) (undenatured) was purchased from Shanghai Yuanye Bio-Technology Co., Ltd. (Catalog No. S12007, Shanghai, China) and was used as a reference. Dual Color protein standard marker (Catalog No. 1610374), 4× Laemmli Sample Buffer (Catalog No. 1610747), and 10× Tris/Glycine/SDS (Catalog No. 1610732) were purchased from Bio-Rad Laboratories Inc. (Hercules, CA, USA). The 5 mM EDTA solution was purchased from Beijing Solarbio Science & Technology Co., Ltd., (Beijing, China). The 0.25% trypsin-EDTA (1×), penicillin/streptomycin (p/s), fetal bovine serum (FBS), and phosphate-buffered saline (PBS, pH 7.4, 1×) were purchased from Thermo Fisher Scientific (Gibco, Waltham, MA, USA). Unless stated otherwise, all reagents were purchased from Sigma-Aldrich Corporation (St. Louis, MO, USA).

### 4.2. Preparation of Collagen-Coated Plates

For the cell proliferation assay, 96-well cell culture plates (3599, Corning, CA, USA) were coated with different concentrations of BSC, SC, and BC in double distilled water, and the plates were air dried below 25 °C. For relative mRNA expression and protein levels of integrin receptor subunits detection, 6-well cell culture plates (3516, Corning, CA, USA) were coated with different collagens, and the drying method is as described above. All plates are sterilized under UV light for 2 h before use. The collagen-coated plates were used in all the experiments except in vitro scratch assay and ECM remodeling tests.

### 4.3. Mouse Bone Marrow-Derived MSCs Culture

Mouse bone marrow-derived MSCs were purchased from Shanghai Zhong Qiao Xin Zhou Biotechnology Co., Ltd. (Shanghai, China) and cultured in mesenchymal stem cell medium (MSCM, Shanghai QiDa Biotechnology Co., Ltd., Shanghai, China) containing 10% FBS, 1% p/s, and 1% mesenchymal stem cell growth supplement (MSCGS), and maintained at 37 °C in a 5% CO_2_ incubator (BB 150, Thermo Fisher Scientific, Waltham, MA, USA). Subsequently, the medium was refreshed every 2 days. When they reached approximately 80% confluence, the cells were detached by trypsinization with 0.25% trypsin-EDTA. The cells from the third to fifth passages were used for the following experiments.

### 4.4. Cell Proliferation Assay

Cell proliferation was evaluated using a Cell Counting Kit-8 (CCK-8; M4839, AbMole, Houston, TX, USA) according to the manufacturer’s instructions. Briefly, a 96-well cell culture plate, mentioned in 4.2, was precoated with the final concentration of 10, 25, 50, 100, or 200 μg/mL collagens, 200 μL solution or suspension (for insoluble collagen type I) per well, respectively. Then, MSCs were seeded in 96-well plates with 5 × 10^3^ cells/well with 200 μL of serum-free MSCM (only supplemented with 1% penicillin/streptomycin and 1% MSCGS) and incubated for 24 h at 37 °C humidified with 5% CO_2_. After incubation, the medium was removed, cells were rinsed using PBS, then 100 μL medium containing 10% CCK-8 reagents was added to each well and incubated for 2 h in a 5% CO_2_ atmosphere at 37 °C without light. At last, absorbance at 450 nm was measured by a microplate reader (BioTek, Winooski, VT, USA). The optical density values were used to assess the cell viability, and control (without adding collagen) was used as the 100% viability group.

### 4.5. In Vitro Scratch Assay

The effect of marine collagens on the migration of MSCs was determined by scratch assay. MSCs were cultured in mesenchymal stem cell medium (MSCM, supplemented with 10% FBS, 1% p/s, and 1% MSCGS) at 37 °C in an atmosphere with 5% CO_2_ before the test. MSCs were trypsinized after reaching a confluence of 90%, seeded into a 24-well cell culture plate (3524, Corning, CA, USA) with 5 × 10^4^ cells/well, and then cultured for 24 h. The 100% cell confluence was reached before the scratch assay was performed. A sterile 200 μL pipette tip was used to make a uniform scratch wound on the monolayer of cells. The medium with cell debris was removed by washing with PBS three times. The scratch monolayer cells were treated with serum-free MSCM containing 100 μg/mL sterile BSC, SC, and BC solution (or suspension). For negative control, cells were cultured in serum-free MSCM without collagen. The images of scratch closure were captured under an optical microscope equipped with a camera (Nikon, Tokyo, Japan) at 0 h, 12 h, and 24 h after incubation with the test collagens. The scratch areas were analyzed by using Image J software https://imagej.nih.gov/ij/index.html (Wayne Rasband, Bethesda, MD, USA), and the wound closure rate (%) was calculated according to the Equation as follows.

$$\mathrm{Wound}\;\mathrm{closure}\left(\%\right)=\frac{\mathrm{Area}\;\mathrm{between}\;\mathrm{cells}\;\mathrm{at}\;0\;h-\mathrm{Area}\;\mathrm{between}\;\mathrm{cells}\;\mathrm{at}\;\mathrm{specified}\;\mathrm{time}}{\mathrm{Area}\;\mathrm{between}\;\mathrm{cells}\;\mathrm{at}\;0\;h}\times 100\%$$

### 4.6. Cell Adhesion Analysis

For cell adhesion studies, a high-binding polystyrene 96-well plate (3361, Corning, CA, USA) was coated with 1 μg per well of BSC, SC, and BC in double distilled water overnight at 4 °C. The non-specific adsorption to the coatings was blocked with 200 μL of 1% bovine serum albumin (BSA, Sangon Biotech Co., Ltd., Shanghai, China) for 1 h at room temperature, and then wells were rinsed with 200 μL of PBS three times. Prior to seeding, MSCs were detached from the cell culture flasks with 0.25% trypsin-EDTA, washed, and resuspended in serum-free MSCM containing either 5 mM MgCl_2_ or 5 mM EDTA. The 8000 MSC cells were seeded into each well for 6 h and allowed to attach at 37 °C humidified with 5% CO_2_. The culture medium was removed, and the wells were thoroughly washed three times with PBS to remove loosely adherent cells. A new culture medium was supplemented, and 10 μL CCK-8 solution was added to each well. The optical density (OD) values were measured at a wavelength of 450 nm through a microplate reader (BioTek, Winooski, VT, USA) and were representative of cell numbers on the collagen coating. Background adhesion was determined on BSA-coated plates. Cell adhesion of BSC in the presence of Mg^2+^ (integrin-mediated) and EDTA (non-divalent cation specific) was evaluated by the varied concentrations from 10 to 125 μg/mL marine collagen, and the experiment was performed according to the method described above. For visualization, wells were washed with PBS after the test; then, cells were fixed with 4% paraformaldehyde (Sangon Biotech Co., Ltd., Shanghai, China) for 15 min and stained with 1 μg/mL DAPI (Invitrogen, Waltham, MA, USA) at room temperature for 10 min. Following the washing steps, the images were captured using a fluorescent microscope fitted with a DP80 camera (IX71, Olympus, Tokyo, Japan).

### 4.7. Cell Spreading Analysis

For spreading analysis, 1 μg BSC, SC, and BC-coated wells were prepared in a high-binding polystyrene 96-well plate (3361, Corning, CA, USA), then BSA blocked surfaces for 60 min at room temperature. Then, 100 μL of cell suspension at 2.5 × 10^4^ cells/mL containing either 5 mM Mg^2+^ in serum-free MSCM was added to each well and incubated at 37 °C/5% CO_2_ for 4 h for MSCs. Cells were fixed after the desired culture time using 4% paraformaldehyde for 15 min at room temperature. The plates were washed 3× with PBS, then 20 μg/mL FITC was added for 10 min at room temperature in the dark, and cell nuclei were stained with DAPI. Representative fluorescence images were obtained using a 20× magnification objective lens on a fluorescent microscope fitted with a DP80 camera. The cell spreading area was calculated from 6–8 representative images by measuring the cell-derived fluorescent area of FITC-stained images in Image J. The cell number for each image was calculated from DAPI-stained images by using the nucleus counter plugin feature of Image J. The average cell area was then calculated by dividing the total cell fluorescent area by the corresponding cell number.

### 4.8. ECM Remodeling Tests

To investigate the interaction of marine collagen-BSC with MSCs in the extracellular matrix, we used a small Alexa Fluor™ 594 NHS Ester (Succinimidyl Ester) (AF594, Invitrogen, Waltham, MA, USA) fluorescent dye (molecular weight: 819.8 Da) to label collagen molecules. AF594 was dissolved in 0.5% dimethyl sulfoxide (DMSO) and then added to the BSC solution to react in 0.2 M sodium bicarbonate buffer (pH 8.3) overnight at 4 °C. The unbound free AF594 dye from labeled BSC was separated using a 50 kDa Merck Millipore ultrafiltration technique (UFC5050BK, Merck, MA, USA). Functionalized BSC molecule probe (AF594-BSC) was washed three times with PBS before characterization and seeding with MSCs, as described below.

#### 4.8.1. Characterization of Functionalized BSC Molecules

Functionalized BSCs were characterized by using sodium dodecyl sulfate-polyacrylamide gel electrophoresis (SDS–PAGE) and circular dichroism (CD). SDS–PAGE analysis was used to determine the molecular pattern of AF594-BSC. Briefly, proteins were mixed with 4 × Laemmli Sample Buffer and were boiled for 5 min. Then, denatured proteins and Dual Color protein standard marker were loaded onto 4.5% stacking polyacrylamide gel with 7.5% separating gel (EpiZyme Biotechnology, Shanghai, China). After the electrophoresis, the gel was stained with Coomassie brilliant blue and discolored until clear protein bands were visualized. The protein bands were captured with ChemiDoc MP Imaging System (Bio-Rad, Hercules, CA, USA).

Circular dichroism (CD) spectra of AF594-BSC were collected from 180 to 300 nm on BRIGHTTIME Chirascan (Applied Photophysics Ltd., Leatherhead, Surrey, UK) using a 1 mm path length cuvette to verify the characteristic CD of the collagen triple-helix at a wavelength of 222 nm. Unlabeled BSC was dissolved in PBS for comparison with collagen molecular probes.

#### 4.8.2. Cell Culture Experiments

MSCs were trypsinized and seeded into a 12-well plate (3513, Corning, CA, USA) at 2 × 10^5^ cells per well and incubated overnight. Growth medium was aspirated from each well and replaced with serum-free MSCM containing the AF594-BSC probe. The culture was imaged using a 40× objective lens on a fluorescent microscope after 6 h and 24 h AF594-BSC addition, and cell membranes were stained with the CellMask green plasma membrane stain (Invitrogen, Waltham, MA, USA), and nuclei with Hoechst 33,342 (Abbkine Scientific, Wuhan, China). This experiment was to perform real-time imaging of extracellular matrix (ECM) development by MSCs supplied with labeled marine collagen molecules.

### 4.9. Quantitative Real-Time Polymerase Chain Reaction(qRT-PCR)

Expression of MSCs integrin subunit genes was evaluated after 24 h cell culture by performing real-time PCR. Total RNA was extracted from MSCs by using the RNA Easy Fast Tissue/Cell Kit (TIANGEN Biotech Co., Ltd., Beijing, China). The RNA concentration was evaluated using micro-spectrophotometry (NanoDrop1000, Thermo Fisher Scientific, Waltham, MA, USA). The extracted mRNA was converted into cDNA using the FastKing RT Kit (TIANGEN Biotech Co., Ltd., Beijing, China) following the manufacturer’s instructions. The gene-specific primers designed to amplify the target genes are provided in Table 1. The expression levels of detected genes were quantified using Talent qPCR Premix (SYBR Green) (TIANGEN Biotech Co., Ltd., Beijing, China) by ABI Applied Biosystems 7500 Real-Time PCR System (Life Technologies, Waltham, MA, USA). Data were analyzed using the 2^−ΔΔCt^ method, and GAPDH was chosen as the housekeeping gene. The results were normalized by the mean values of the corresponding control groups.

### 4.10. Enzyme-Linked Immunosorbent Assay (ELISA)

Protein expression of integrins in the MSCs cultured with marine collagens was detected by ELISA kits (MEIMIAN, Yancheng, China), according to the instructions of the manufacturer. In brief, MSCs were inoculated into 6-well culture plates, as described in 4.2, with 10^5^ cells per well, followed by the addition of 1 mL serum-free MSCM for 24 h culture. At the end of the culture, cells were disrupted with cell lysate (Absin, Shanghai, China) and centrifuged to harvest the supernatant of MSCs. Then, the protein concentrations were quantified using a BCA Protein Assay Kit (TIANGEN Biotech Co., Ltd., Beijing, China). Subsequently, 50 uL of the resulting supernatant was, respectively, added to the integrin α_1_β_1_, α_2_β_1_, α_10_β_1_, α_11_β_1_ antibody-coated microplate for incubation at 37 °C for 30 min, whereupon they were repeatedly washed with 300 uL of washing solution for five times. Following the instructions, 50 uL of corresponding horseradish peroxidase (HRP)-labeled integrin antibody was added for incubation at 37 °C for 30 min. After thorough washing, TMB chromogen solution was added to each well, evading the light preservation for 10 min at 37 °C. After the termination of the reaction, the optical density (OD) at a wavelength of 450 nm was measured by a microplate reader (BioTek, Winooski, VT, USA). Photometric values were quantified and normalized to the control group (without adding collagen).

### 4.11. Statistical Analysis

All data are expressed as mean ± standard deviation (SD) of three independent replicates and mentioned in the figure legends. Statistical analysis was performed with GraphPad Prism 9 (GraphPad Inc., San Diego, CA, USA) using unpaired two-tailed student’s *t*-test and a two-way ANOVA with Tukey’s multiple comparison analysis to determine significant differences between groups. The values identified as outliers were excluded from the statistical analysis. A *p* value < 0.05 was considered statistically significant.

## 5. Conclusions

In summary, we found that marine-derived BSC potentially affected the cellular behavior of mesenchymal stem cells. More specifically, we demonstrated that the BSC and SC significantly promoted the cell processes such as proliferation, migration, adhesion, and spreading through the collagen-integrin binding interactions. Our data showed that the observed differences in cell response might result from the high expression of integrin receptors directly bound with collagen. We also investigated the remodeling of the ECM by observing the collagen deposition in the cell microenvironment using live cell imaging. Consequently, this work concluded that blacktip reef shark skin collagen could be a potential biomaterial to support stem cell-based applications in tissue engineering and regenerative medicine.

## Figures and Tables

**Table 1:** Primer sequences used for qRT-PCR procedures.

Gene	Primer	Sequence	Tm (°C)	GenBank
ITGA1	Forward	5′-CACTGATCTGCTTCTCGTCGG-3′	60.80	NM_001033228.3
	Reverse	5′-CTGATTCACAGCGTACACGTA-3′	58.14	
ITGA2	Forward	5′-GGGGACCTCACAAACACCT-3′	59.16	NM_001033228.3
	Reverse	5′-CAGTTTTCAGCTTCGACCCAT-3′	58.85	
ITGA10	Forward	5′-GCTTCTCCATCCACCGACT-3′	59.10	NM_001302471.1
	Reverse	5′-ACCTTCTTCAAGCCATAGCAC-3′	58.28	
ITGA11	Forward	5′-GGCACCAACAAGAATGAGACC-3′	59.46	NM_176922.5
	Reverse	5′-CCCCGTTCCAGTCATAGGC-3′	59.85	
ITGB1	Forward	5′-GCACACTGTCTGGAAACTCT-3′	57.75	NM_010578.2
	Reverse	5′-TTGTTACTCCGTCTGGCAAT-3′	57.15	
GAPDH	Forward	5′-TCAACGACCCCTTCATTGACC-3′	60.27	NM_008084.3
	Reverse	5′-ACTGTGCCGTTGAATTTGCC-3′	59.97	

## Data Availability

Not applicable.
